# Isolated Right Ventricular Dilated Cardiomyopathy: An Early Diagnosis

**DOI:** 10.14740/jocmr2165w

**Published:** 2015-08-23

**Authors:** Sem Briongos Figuero, Alvaro Acena Navarro

**Affiliations:** a Cardiology Department, Infanta Leonor Hospital, Madrid, Spain; b Cardiology Department, Foundation Jimenez Diaz Hospital, Madrid, Spain

**Keywords:** Right ventricle, Dilated cardiomyopathy, Idiopathic

## Abstract

Because of an incomplete right bundle branch block, a severe right ventricular dilatation with no left ventricular cardiomyopathy was found in a 44-year-old man. Magnetic resonance and transesophageal echocardiography confirmed the finding and these tests also failed to find any potential cause. A pulmonary hemodynamic study and a coronary angiography were strictly normal. Lastly pulmonary function tests and a pulmonary angiography were performed, which did not find any lung disease causing the right ventricular dilatation. The patient was catalogued as an early stage of an idiopathic form of right ventricular dilated cardiomyopathy.

## Introduction

The etiologic diagnosis of right ventricular dilatation remains a diagnostic challenge. Many causes have been shown implicated in its onset. A careful step-by-step approach is necessary to reach the final diagnosis of idiopathic right ventricular dilated cardiomyopathy. The striking about our report is the description at an early stage we did, after an exclusion of any possible secondary cause.

## Case Report

**Figure 1:**
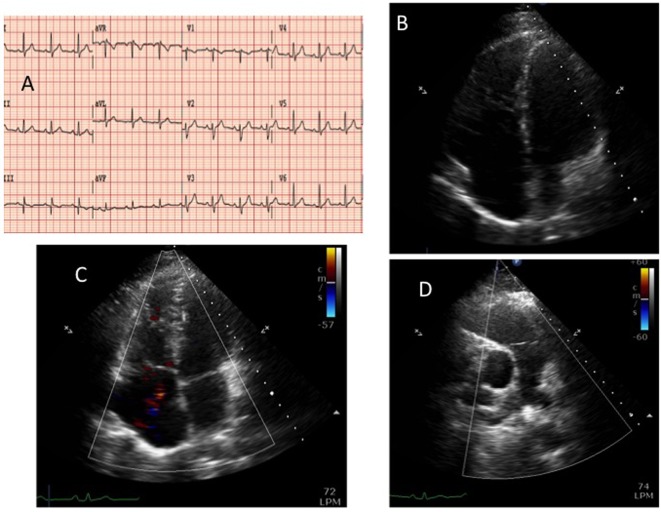
The 12-lead electrocardiogram (panel A). Transthoracic echocardiography: apical four-chamber view, diastolic frame (panel B) showing a severe dilatation of the right ventricle and color-Doppler systolic frame (panel C). Short axis parasternal view (panel D): dilatation of the right ventricle outflow tract but no dilatation in the pulmonary artery.

**Figure 2:**
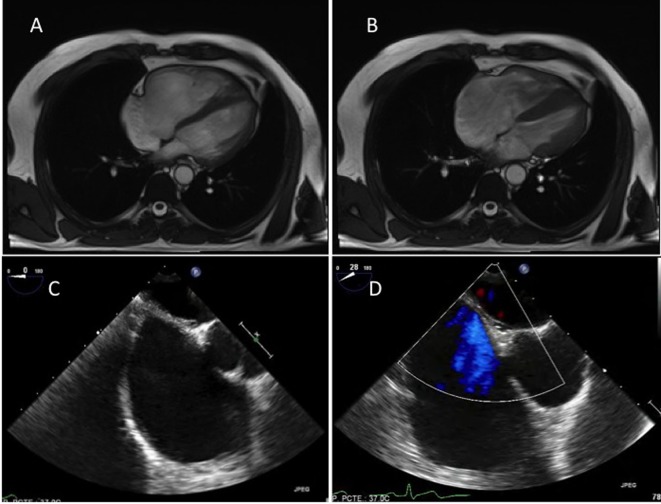
Magnetic resonance, four-chamber view: diastolic (panel A) and systolic (panel B) frame, exhibiting normal right ventricular shortening. Transesophageal echocardiography, mid-esophageal plane (panel C) and also a color-Doppler (panel D) view exhibiting no interatrial shunt.

A 44-year-old man was referred to the cardiology department because of an incomplete right bundle branch block (IRBBB) detected in the electrocardiogram (EKG). No epsilon or negative T waves were found on the EKG (Fig. 1). He had no history of familiar sudden cardiac death and he did not have any previous disease despite he was a carrier of C-hepatitis virus. No medication was needed for the treatment of that virus because transaminase enzymes in blood analysis were in normal range and abdominal echography showed normal liver morphology. He was only taking sertraline and bromazepam because of depressive syndrome. He remained completely asymptomatic, in NYHA functional class I/IV, and his physical examination did not show any abnormal finding. In order to begin the study of the IRBBB we first performed an echocardiogram in which a severe right ventricle (RV) dilatation (Fig. 1) and a moderate tricuspid regurgitation (TR) appeared. Left ventricle ejection fraction (LVEF) was preserved (55%) and mitral and aortic valves were normal, in function and morphology. The estimated systolic pulmonary pressure was normal and there was not any indirect sign of pulmonary hypertension. To better characterize the findings in the RV, a magnetic resonance (MR) was performed. It showed a diastolic diameter of the RV of 55 mm (Fig. 2) and confirmed the functional cause of the TR (ring dilatation). There were not any dyskinesia or akinesia areas (Supplementary Videos 1 and 2, www.jocmr.org) or pathologic gadolinium enhancement, and RV ejection fraction was 57%. Abnormal venous drainage was also excluded with the MR. The transesophageal echocardiography did not find any interatrial (Fig. 2) or interventricular septal defects. Holter monitoring did not find any arrhythmic event. Hemodynamic study showed normal pulmonary pressures (systolic pulmonary artery pressure/mean pulmonary artery pressure/diastolic pulmonary artery pressure of 24/14/7 mm Hg respectively) and normal pulmonary wedge pressure (12 mm Hg) which led us to exclude pulmonary hypertension as the cause of the RV dilatation. Cardiac ischemic disease was excluded by performing a coronary angiography. No atherosclerosis plaques in the coronary arteries were found. Pulmonary functions tests (forced expiratory volume in 1 s (FEV1): 117%; FEV1/forced vital capacity (FVC): 84%), diffusion capacity (DLCO: 115%), artery gasometry and polysomnographic study were also normal. We lastly performed a pulmonary angiography, in which a chronic thromboembolic pulmonary disease was also excluded. We decided not to start any medication due to the lack of symptoms of the patient and we also decided to keep a watchful waiting attitude. The patient was catalogued as an isolated form of right ventricular dilated cardiomyopathy due to the finding of an RV dilatation, without any potential cause for it. After 2 years of follow-up, the patient remains asymptomatic and the RV persists dilated.

## Discussion

The first cases reported in the literature suggesting the possibility of an idiopathic form of RV cardiomyopathy were published in the decade of the eighties [1, 2]. The development of the imaging techniques redefined these initial patients and confirmed that these early reports corresponded to people affected by arrhythmogenic RV cardiomyopathy (ARVC). There are only a few cases of true idiopathic RV dilated cardiomyopathy (IRVDM). Most of them have been diagnosed by an autopsy, because of a fatal clinical course with severe heart failure and arrhythmias [3, 4]. Our patient does not fulfil criteria for ARVC: he only had RV dilatation but without any dyskinesia or akinesia area, the EKG only showed an IRBBB but without epsilon or T negative waves, no familiar history related to ARVC was found and there were not ventricular arrhythmic events. So we can conclude that this is the first form of IRVDM described in an early phase of the disease, after any potential cause was excluded. A cardiac biopsy would have achieved a better characterization of this disease but the potential risk of this technique is probably higher than the benefit, especially taking into account the lack of symptoms of the case. A close follow-up is mandatory in order to improve our knowledge of this rare entity and to treat as soon as possible the potential complications reported in the previous cases of this disease.
